# Autistic Adults Avoid Unpredictability in Decision-Making

**DOI:** 10.1007/s10803-024-06503-2

**Published:** 2024-08-19

**Authors:** Ana Macchia, Laura Albantakis, Paul Theo Zebhauser, Marie-Luise Brandi, Leonhard Schilbach, Anna-Katharine Brem

**Affiliations:** 1 Max Planck Institute of Psychiatry, Munich, Germany; 2 Clinic for Psychiatry/Psychotherapy III, Ulm University, Ulm, Germany; 3 Independent Max Planck Research Group for Social Neuroscience, Max Planck Institute of Psychiatry, Munich, Germany; 4 International Max Planck Research School for Translational Psychiatry, Munich, Germany; 5 Department of Psychiatry and Psychotherapy, Ludwig Maximilians University, Munich, Germany; 6 School of Medicine, Department of Neurology, Technical University of Munich, Munich, Germany; 7 Department of General Psychiatry 2, LVR-Klinikum Duesseldorf/Kliniken der Heinrich-Heine-Universitaet, Duesseldorf, Germany; 8 Ludwig-Maximilian-University, Munich, Germany; 9 University Hospital of Old Age Psychiatry, University of Bern, Bern, Switzerland; 10 Centre for Healthy Brain Ageing, Department of Psychological Medicine, Institute of Psychiatry, Psychology and Neuroscience, King’s College London, London, UK

**Keywords:** Autism spectrum, Decision-making, Iowa gambling task, Cambridge risk task, Trail making test, Hormones

## Abstract

Decision-making under unpredictable conditions can cause discomfort in autistic persons due to their preference for predictability. Decision-making impairments might furthermore be associated with a dysregulation of sex and stress hormones. This prospective, cross-sectional study investigated decision-making in 32 autistic participants (AP, 14 female) and 31 non-autistic participants (NAP, 20 female) aged 18–64 years. The Iowa Gambling Task (IGT) and the Cambridge Risk Task (CRT) were used to assess decision-making under ambiguity and under risk with known outcome probabilities, respectively. Cortisol, estradiol, and testosterone serum levels were related to decision-making performance. Groups did not differ in overall IGT and CRT performance, but compared with NAP, AP preferred less profitable card decks with predictable outcomes while avoiding those with unpredictable outcomes. AP required more time to reach decisions compared to NAP. Additionally, AP without comorbid depression performed significantly worse than NAP in the IGT. Estradiol and cortisol concentrations were significant predictors of CRT scores in NAP, but not in AP. The study results imply that AP are ‘risk-averse’ in decision-making under ambiguity as they avoided choice options with unpredictable losses in comparison to NAP. Our findings highlight the intolerance for uncertainty, particularly in ambiguous situations. Thus, we recommend being as transparent and precise as possible when interacting with autistic individuals. Future research should explore decision-making in social situations among individuals with ASD, factoring in person-dependent variables such as depression.

**Supplementary Information:**

The online version contains supplementary material available at 10.1007/s10803-024-06503-2.

In everyday life, human beings are regularly confronted with unpredictable situations. For autistic individuals these situations may be especially challenging given their tendency for predictability, adherence to habits and inflexibility towards change (Bora & Pantelis, 2016). Although these preferences might be more pronounced in social situations with high degrees of uncertainty, they might also manifest in situations of non-social decision-making (Allman et al., 2005; Ruff & Fehr, 2014).

Decision-making is a component of executive functioning, involving various cognitive processes such as memory, planning, reasoning, judging, response anticipation, and response inhibition (Del Missier et al., 2012; Swami, 2013). Nevertheless, it has been widely accepted that emotions are used to modulate decision-making (Damasio, 1996; Lerner et al., 2015). Both, executive dysfunctions like cognitive inflexibility, as well as deficits in metacognition including alexithymia (i.e., difficulties in identifying and describing emotions) are common in autistic individuals (Albantakis et al., 2020; Wallace et al., 2016), and most likely affect decision-making. In fact, autistic adults experience difficulties with decision-making, especially when decisions need to be made quickly or involve a change of routine (Demetriou et al., 2018; Luke et al., 2012). Risk-aversion and enhanced rationality are being discussed as key features of decision-making in autism, and as possible strengths of autistic individuals (Brosnan et al., 2016; Gosling & Moutier, 2018; Vella et al., 2018). Studies to date, however, report inconsistent findings with some studies describing more rational decision-making in autistic people (South et al., 2014; Vella et al., 2018), and others observing less rational behavior (Mussey et al., 2015; Zhang et al., 2015). Interestingly, Robic et al. (2015) did not find any differences between non-autistic comparison participants (NAP) and autistic participants (AP) in predictable conditions, but observed difficulties in decision-making under unpredictable conditions in AP.

This finding contributes to the assumption that core phenotypic symptoms of autism, like insistence on sameness and repetitive behavior, derive from compromised prediction skills that may be particularly important during social interactions (Bolis et al., 2017; Sinha et al., 2014). Moreover, unexpected outcomes affect emotional responses more strongly than expected outcomes (Mellers et al., 1997). Thus, ambiguous and highly complex situations, which pose a challenge to correctly predict an outcome, might reveal decision-making deficits in AP. De Martino and colleagues (2008) propose that autistic individuals use higher logical consistency in decision-making under uncertainty because of enhanced analytical tendency and the failure to perceive and integrate emotional cues into the decision-making process. Potential modulators of decision-making under ambiguity and risk-taking are stress and sex hormones (Ambrase et al., 2021; Derntl et al., 2014; Kurath & Mata, 2018; Sarmiento Rivera & Gouveia, 2021). More precisely, elevated (basal) levels of cortisol, estradiol, and testosterone have been associated with increased risk-taking and a higher probability for conflict, fear, stress, and threat in both men and women (Ambrase et al., 2021; Carney & Mason, 2010; Herbert 2018). Moreover, testosterone interacts with cortisol to predict risk-taking (e.g., Mehta et al., 2015). Although recent data in autism research indicates that timing is critical for the effect of sex hormones on the brain with focus on the prenatal rather than the postnatal period (Baron-Cohen et al., 2020), it is possible that - like in NAP – steroidogenic activity might affect decision-making in autistic individuals.

This study investigated decision-making in AP and its putative relationship with hormone levels. We examined decision-making under ambiguity (low outcome predictability, high ambiguity) and under risk with known outcome probabilities (high outcome predictability, low ambiguity). We hypothesized that AP show impaired decision-making, when facing high but not low ambiguity and explored if hormone levels interact with decision-making in both groups.

## Methods

### Sample

A total of 63 out of 68 participants (age range 18–64 years, *N* = 31 NAP [20 females, 64.52%] and *N* = 32 AP [14 females, 43.75%]), who participated within the framework of another study (Albantakis et al., 2021) at the Outpatient and Day Clinic For Disorders of Social Interaction and the independent Max Planck Research Group for Social Neuroscience at the Max-Planck Institute of Psychiatry in Munich, Germany (data collection November 2017-March 2019), took part in this sub-study. The eligibility criteria were no hormonal deficiencies or stable substitution thereof, intelligence quotient above 70. The exclusion criteria comply with those of the main study (Albantakis et al., 2021) and consisted of any serious somatic illness (e.g. diabetes, diseases of the cardiac, pulmonary, renal or hepatic system, chronic inflammatory diseases etc.), IQ values below 70, a diagnosis of schizophrenia in the present or past, breast-feeding, pregnancy, and the use of hormonal contraception and/or sex hormones. Only individuals who met the DSM-5-criteria for autism spectrum disorder (APA, 2013), following an extensive clinical assessment, were admitted to the study (for detailed procedures see Albantakis et al., 2018, 2021). NAP were defined as adults without any history of psychiatric or neurological disorders. More than half of the AP had a comorbid diagnosis of depression (*n* = 17, 53.13%) and one participant had an attention deficit hyperactivity disorder as secondary diagnosis (03.13%). Many autistic participants with comorbid depression were additionally affected by another psychiatric comorbidity (*n* = 7, 21.88%) including anxiety disorders (e.g., social phobia), obsessive-compulsive disorders, and behavioral and emotional disorders with onset usually occurring in childhood and adolescence (attention deficit hyperactivity disorder, Tic-disorder). Five participants (*n* = 3 NAP, *n* = 2 AP) were excluded due to other serious medical conditions (*n* = 3), medication intake (*n* = 1) or aggressive behavior during testing (*n* = 1). Due to technical issues during the CRT (*n = 4*), 29 NAP and 30 AP were enrolled in the CRT analysis. For the hormonal analyses, blood samples from two AP (one female and one male) had to be excluded as their estradiol levels exceeded the sex specific reference range. Thus, blood samples from 29 NAP and 27 AP were analyzed. 50% of the AP took psycho-pharmaceuticals (*n* = 16) and were asked to take their regular medication after the study visit (Albantakis et al., 2021) (Supplement Table S1). Table 1 shows clinical characteristics of the sample. Ethical approval was granted by the Ethics Committee of the Ludwig-Maximilians-University (LMU) Munich (Project number: 712–15). All procedures were performed in accordance with the Declaration of Helsinki. Participants could withdraw from the study at any time and were financially compensated with 10 euros per hour.

**Table 1:** Clinical characteristics of the study sample

	NAP (*N* = 31)		AP (*N* = 32)			
	*M*	*SD*	*M*	*SD*	*t/ U*	*p*
Age (years)	31.03	11.73	36.38	10.45	335.5 ^a^	0.0271*
AQ score	13.65	5.30	34.80	12.16	57.62	< 0.001***
BDI-II score	4.48	5.15	13.47	11.68	-3.97	< 0.001***

*Note* NAP: Non-autistic comparison participants; AP: Autistic participants; AQ: Autism-spectrum quotient; BDI-II: Beck Depression Inventory-II (scale: 0–63)

^a^ In NAP, age did not follow a normal distribution (Supplement Table S2-S3). Significance of parametric and non-parametric methods were not consistent. Results are based on the Mann-Whitney-U-test.

* Indicates *p* < .05. ** indicates *p* < .01. *** indicates *p* < .001

### Neuropsychological Tests

#### Iowa Gambling Task (IGT)

The IGT is a computerized task consisting of 100 trials. The object of the game-like task is to increase initial game money of $2,000 by selecting cards from four card decks that differ in payoff features. The two profitable or less risky decks (“good card decks”) lead to small immediate wins ($50) with occasional smaller losses (deck C: $50, deck D: $250). The two risky card decks show a reverse pattern (“bad card decks”). High immediate payoffs ($100) and high losses (deck A: $150- $350, deck B: $1,250) lead to an overall less profitable outcome. Each time participants choose a card, they receive feedback on the outcome (win or loss) and the running game money. Moreover, the internal features of “good” and “bad” decks differ in loss and win frequency. Win frequency is high for decks B and D and low for decks A and C. As the game progresses, NAP usually figure out the implicit reward features of the game and increasingly choose “good card decks” (decks C and D), resulting in a higher total reward (Brand et al., 2007). The IGT net score is used as indicator of task performance and is calculated as a difference score between the overall proportion of good and bad decks (net score = advantageous decks – disadvantageous decks).

#### Cambridge Risk Task (CRT)

The CRT was designed to assess decision-making under certain risk conditions (i.e., magnitudes and probabilities of gains and losses are known). An array of six colored boxes is presented on a computer screen and appears at varying ratios (1/6, 2/6, 3/6, 4/6, 5/6) of red and blue boxes on each trial. The participants’ task is to maximize their initial score of 100 by deciding whether a token is hidden behind a red or blue box. Upon correctly locating the token, the total point score increases, while it decreases after incorrect choices by a given amount associated to the choice of the red or blue box, respectively (red/blue – 10/90; 20/80; 30/70; 40/60). Accordingly, selection of a color that is underrepresented increases the risk but is also rewarded higher. After each selection, auditory feedback is provided by a rising musical tune for wins and a low-pitched tone for losses. Decision-making measures include (1) overall bet proportion: higher points indicate preference for putting more points at risk (mean score obtained across all trials); (2) deliberation time (mean choice reaction time); (3) risky/rational choices: higher scores indicate more rational choices (number of choices of the more probable option, note that the equal risk ratio is not meaningful for low risk choices).

#### Questionnaires

Autistic traits were measured with the Autism-Spectrum Quotient (AQ), a self-administered questionnaire designed for adults with average intelligence (Baron-Cohen et al., 2001). It consists of 50 statements, each allowing the subject to indicate the degree of agreement with it (“definitely agree”, “slightly agree”, “slightly disagree”, “definitely disagree”). Depressive symptoms were assessed using the Beck Depression Inventory (BDI-II), which comprises 21 items with possible scores ranging from 0 to 3 per item (Beck et al., 1996).

#### Quantification of Hormones

For the quantification of cortisol plasma was used. First, blood was taken into blood collection tubes (Sarstedt, S-Monovette K3E 2.7 ml or 7.5 ml, Cat.nr.: 01.1605.001). and transported in a cooling box immediately after blood collection. Then blood was centrifuged at 4 °C for 15 min with 2500 x g. The supernatant was filled into a 4 ml tube (Sarstedt, 92 × 15.3 mm, PP, Cat.nr.: 62.611) and centrifuged again. After centrifugation the samples were aliquoted into 2D-barcode tubes (Brooks, fluidX, Cat.nr.: 68-0703-12) and stored at -80 °C (Albantakis et al., 2021).

For the quantification of estradiol and testosterone serum was used. First, blood was taken into blood collection tubes (Sarstedt, S-Monovette, 92 × 15 mm, 7.5 ml, Cat.nr. 01.1602.001). All specimens were allowed to clot for 30 min at room temperature. Then they were centrifuged at 4 °C for 15 min with 2500x g. After centrifugation the samples were aliquoted into 2D-barcode tubes (Brooks, fluidX, Cat.nr.: 68-0703-12) and stored at -20 °C for the first 24 h and then at -80 °C.

Plasma cortisol was determined by using an Enzyme-linked Immunosorbent Assay (*ELISA*) kit (RE52061, TECAN, IBL Hamburg, Germany). The Standard Range was 20–800 ng/ml. The analytical sensitivity (limit of detection) is 2.46 ng/ml, the 2 SD functional sensitivity is 4.03 ng/ml and the mean concentration is < 20% CV; cross-reactivity of other substances tested < 0.01%; intra-assay < 3.48; inter-assay < 3.42 (Albantakis et al., 2021).

Serum estradiol was determined by using an Enzyme-linked Immunosorbent Assay (ELISA) kit (RE52041, TECAN, IBL Hamburg, Germany). The Standard Range was 9.7–2000 pg/mL. The Analytical Sensitivity (Limit of Detection) is 10.6 pg/mL, and the Intra- Assay Mean Conc. is < 9.2% CV, Inter-Assay < 14.9% CV.

Serum testosterone was determined by using an enzyme-linked immunosorbent assay (ELISA), kit (RE52151, TECAN, IBL Hamburg, Germany). The Standard Range was 0.2–16 ng/mL. The Analytical Sensitivity (Limit of Detection) is 0.12 ng/mL. The Functional Sensitivity is 0.18 ng/mL with the Mean Conc. <20% CV. The Intra- Assay Mean Conc. is < 5.4% CV, Inter- Assay < 7.4% CV.

### Design

Neuropsychological testing was performed on one of two study days either in the morning (9:00 am) or after a break of one hour from another task unrelated to the present study (12:00 pm). During the break participants were allowed to drink and eat. Blood samples were collected in the morning of day two between 9.00 am and 9.45 am after a fasting state (> 12 h), as described in more detail elsewhere (Albantakis et al., 2021).

The IGT and the CRT were performed consecutively for a total time of approximately 30 min. Instructions of the decision-making tasks were presented in English on the computer screen and the German translation on a separate sheet.

### Statistical Analyses

Data processing and statistical analyses were performed in RStudio (R Core Team, 2023), IBM SPSS 25.0 (IBM Corp., Armonk, NY, USA), and Matlab (R2010a, The MathWorks, Inc., Natick, MA, USA). We used the ‘lme4’ R-package (Bates et al., 2018) to analyze all repeated measure with linear mixed-effects models. The models were analyzed via restricted maximum likelihood and included random-effects of participants. To explore task performance of the IGT (IGT net score) and alternating choice behavior (deck level preferences [A, B, C, D]), group (dummy coded: NAP = 0, AP = 1), block (treated as continuous variable [1 to 5; 20-trial block]) and the group x block interaction were included as fixed-effects. Speed of decision-making and low risk choices (i.e., more probable outcome chosen) of the CRT were analyzed including fixed-effects of group, risk ratio (treated as continuous variable [1 to 6]), and the group x risk ratio interaction. *P*-values for regression coefficients were calculated using the ‘lmerTest’ package (Kuznetsova et al., 2017), which applies Satterthwaite’s approximation to estimate denominator degrees of freedom. We provide detailed information about linear mixed-effects models in the Supplement (Table S7 - S21). The influence of group on outcomes of non-repeated measure data of the CRT overall bet proportion (mean score obtained across all trials) was examined with linear regression. We conducted subsample analyses to explore the impact of mood on IGT performance by including the BDI-II score into the model and by dividing the groups into AP with and without comorbid depression (dummy coded: NAP = 0, AP = 1, AP with depression = 2). Since clinical characteristics of the sample differed between groups, age (mean centered) and sex (dummy coded: 0 = male, 1 = female) were entered as covariates into each model to control for their potential influence.

We checked each model for (1) normality of residuals, (2) linearity between predictor and response, (3) homoscedasticity, (4) multicollinearity, and (5) influential data points. For the deck level preference of deck C of the IGT, overall bet proportion of the CRT, deliberation time of the CRT, and low risk choices of the CRT visual inspection of residual plots indicated violations of testing assumptions. If not explicitly mentioned in the text, the models on transformed data did not result in changes of statistical significance. The variance inflation factors (VIFs) ranged from 1.04 to 3.60, suggesting that the models were not affected by multicollinearity. Cook’s distance was used to examine influential data points. One participant was excluded as a general outlier due to knowing the task structure (i.e., most profitable deck) of the IGT (*SD* > 2.5). We used a rule of thumb (see Nieuwenhuis et al., 2012 for details) to consider a value as too influential (cut-offs: IGT ≤ 0.065; CRT ≤ 0.068). Models were re-evaluated successively after the exclusion of influential data points using the ‘influence.ME’ (Nieuwenhuis et al., 2012) package in R. The exclusion of influential cases resulted in changes of statistical significance for the IGT net score. Additional models without influential data points are provided in the Supplement. The threshold of statistical significance was set to *p* < .05.

### Hormones and Decision-Making Behavior

Cortisol concentrations were normally distributed among NAP and AP (Shapiro-Wilk, *p-value* > 0.05), while estradiol and testosterone concentrations were not. To achieve a normal distribution, we applied the “Ordered Quantile (ORQ) normalization transformation” for estradiol and testosterone measures, which was suggested by the R package “BestNormalize” (Peterson & Peterson, 2020).

Linear regression analyses were performed in SPSS to reveal associations between the behavioral phenotype and hormonal status, namely cortisol, estradiol, and testosterone concentrations at baseline. First, backwards selection was applied to identify the most parsimonious model by focusing on the *F*-statistic of each model and the *p*-values of the entered variables. This approach allowed us to observe the relative impact of the different hormones on the outcome of the neuropsychological tasks, even if they were not included in the final models. Cortisol, estradiol, and testosterone concentrations were included in the model as independent variables. To control for potential age and sex effects, both variables were additionally entered as independent variables, while IGT net score and transformed CRT score (root transformation see Supplement) were included as dependent variables, respectively. Second, to provide more robust statistics, we applied 1,000 resamples bootstrapping with bias-corrected and accelerated confidence intervals (BCa Cis) in the linear regression models (Field et al., 2012).

## Results

### Decision-Making as Measured with the IGT

#### IGT Net Score

**Fig. 1:**
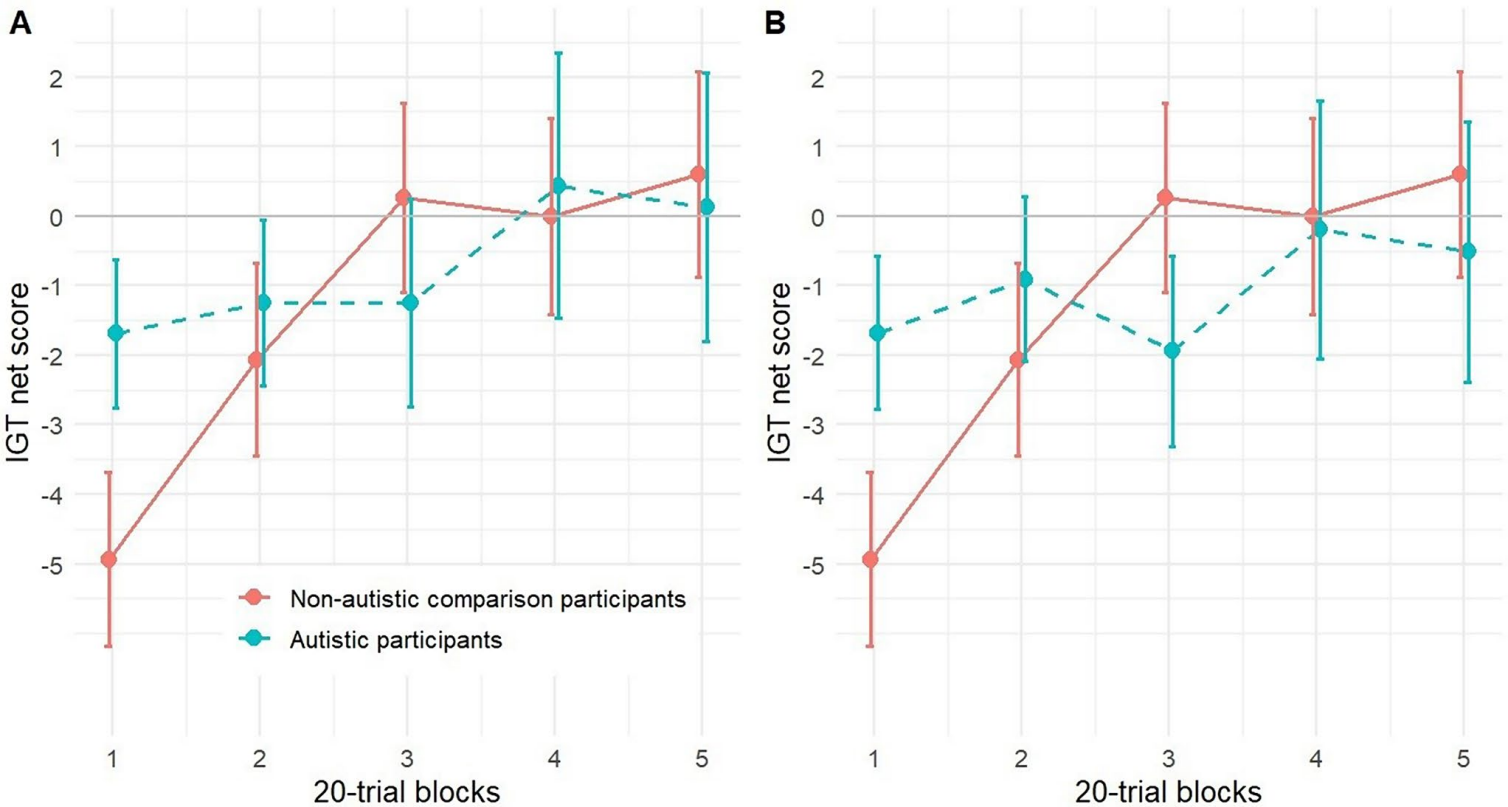
The mean IGT net score of NAP and AP. *Note*. The IGT net score (advantageous decks – disadvantageous decks) (y-axis) for each block of the IGT consisting of 20 trials (x-axis) with (Figure **A**, *N* = 62) and without the influential case (Figure **B**, *N* = 61). Error bars indicate standard errors

We found a significant relationship between block and IGT score (*b* = 1.31, 95% CI [0.60, 2.02], *t*(246) = 3.62, *p* < .001; β = 0.23, 95% CI [0.10, 0.35]), such that later blocks were associated with a higher IGT score. There was no main effect of group, and the interaction effect of block x group was not significant. None of the other predictors of this model (age, sex) were of statistical significance. There was a significant interaction effect of block x group after the exclusion of an influential case (*b* = -1.01, 95% CI [-1.97, -0.05], *t*(242) = -2.06, *p* = .040; β = -0.18, 95% CI [-0.35, -0.01]), showing that AP tended to have lower IGT net scores in later blocks in comparison with NAP (Fig. 1) (Supplement Table S4 -S5, S7-S8). This indicates that the model was strongly influenced by a single case, who was an autistic individual quickly figuring out the task structure and achieving the highest possible IGT net score (= 20) in blocks three to five. Subsample analyses indicated a significant block x group interaction (*b* = -1.61, 95% CI [-2.84, -0.38], *t*(245) = -2.58, *p* = .011; β = -0.28, 95% CI [-0.49, -0.06]), showing that only AP without comorbid depression performed significantly worse in later blocks compared to NAP (Supplement Table S9). The incorporation of the BDI-II score into the model did not yield a significant correlation between the level of depression and the IGT score (Supplement Table S10).

#### IGT Deck Level Preference

**Figs. 2:**
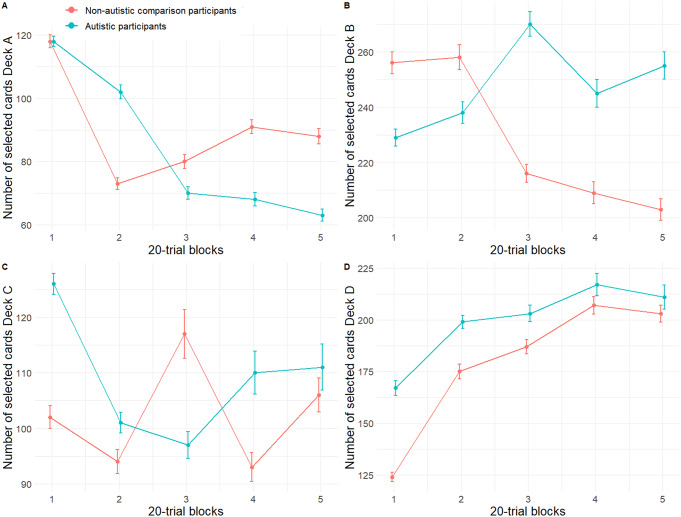
The number of selected card decks in the IGT of NAP and AP (**a-d**). *Note.* The number of selected card decks in the IGT for deck A (“bad deck”, infrequent gains), deck B (“bad deck”, frequent gains), deck C (“good deck”, infrequent gains) and deck D (“good deck”, frequent gains) of NAP (y-axis). The number of selected card decks is presented for each block of the IGT consisting of 20 trials (x-axis). Error bars indicate standard deviations

There was a significant group x block interaction for deck A (“bad deck”, infrequent gains) (*b* = -0.31, 95% CIs [-0.59, -0.03], *t*(246) = -2.18, *p* = .030, β = -0.21, 95% CI [-0.39, -0.02]). AP chose deck A less frequently across the trials compared to NAP (Fig. 2a). Main effects of group, block, and the covariates (age, sex) did not significantly contribute to the model (Supplement Table S11). For deck B (“bad deck”, frequent gains) we found a significant, negative effect of group (*b* = -2.34, 95% CI [-4.54, -0.13], *t*(58) = -2.08, *p* = .039, β = -0.06, 95% CI [-0.45, 0.34]) and block (*b* = -0.52, 95% CI [-0.87, -0.16], *t*(246) = -2.88, *p* = .004, β = -0.18, 95% CI [-0.30, -0.06]). Moreover, there was a significant group x block interaction (*b* = 0.70, 95% CI [0.21, 1.19], *t*(246) = 2.81, *p* = .005, β = 0.24, 95% CI [0.07, 0.41]), indicating that, as opposed to NAP, AP chose deck B more frequently throughout the blocks (Fig. 2b; Supplement Table S12). None of the main block- or interaction effects for the number of cards chosen from deck C (“good deck”, infrequent gains) was significant, nor did any of the covariates significantly influence the model (Fig. 2c, **S**upplement Table S13, S14). There was a significant effect of block (*b* = 0.63, 95% CI [0.29, 0.98], *t*(246) = 3.60, *p* < .001, β = 0.22, 95% CI [0.10, 0.34]) on the number of cards chosen from deck D (“good deck”, frequent gains). This suggests that NAP and AP chose more cards from deck D in later blocks (Fig. 2d). None of the other main or interaction effects were statistically significant (Fig. 2; Supplement Table S15).

### Decision-Making as Measured with the CRT

#### Overall Bet Proportion

The model was not statistically significant, *R*² = 0.03, *R*² adjusted = − 0.02, *F*(3, 55) = 0.65, *p* = .587. There was no significant influence of group (β = − 0.07, *p* = .791, 95%CI [-0.63, 0.49]), sex (β = − 0.02, *p* = .439, 95%CI [-0.77, 0.34]) or age (β = − 0.12, *p* = .396, 95%CI [-0.41, 0.16]) on the overall bet proportion of the CRT.

#### Deliberation Time

**Fig. 3:**
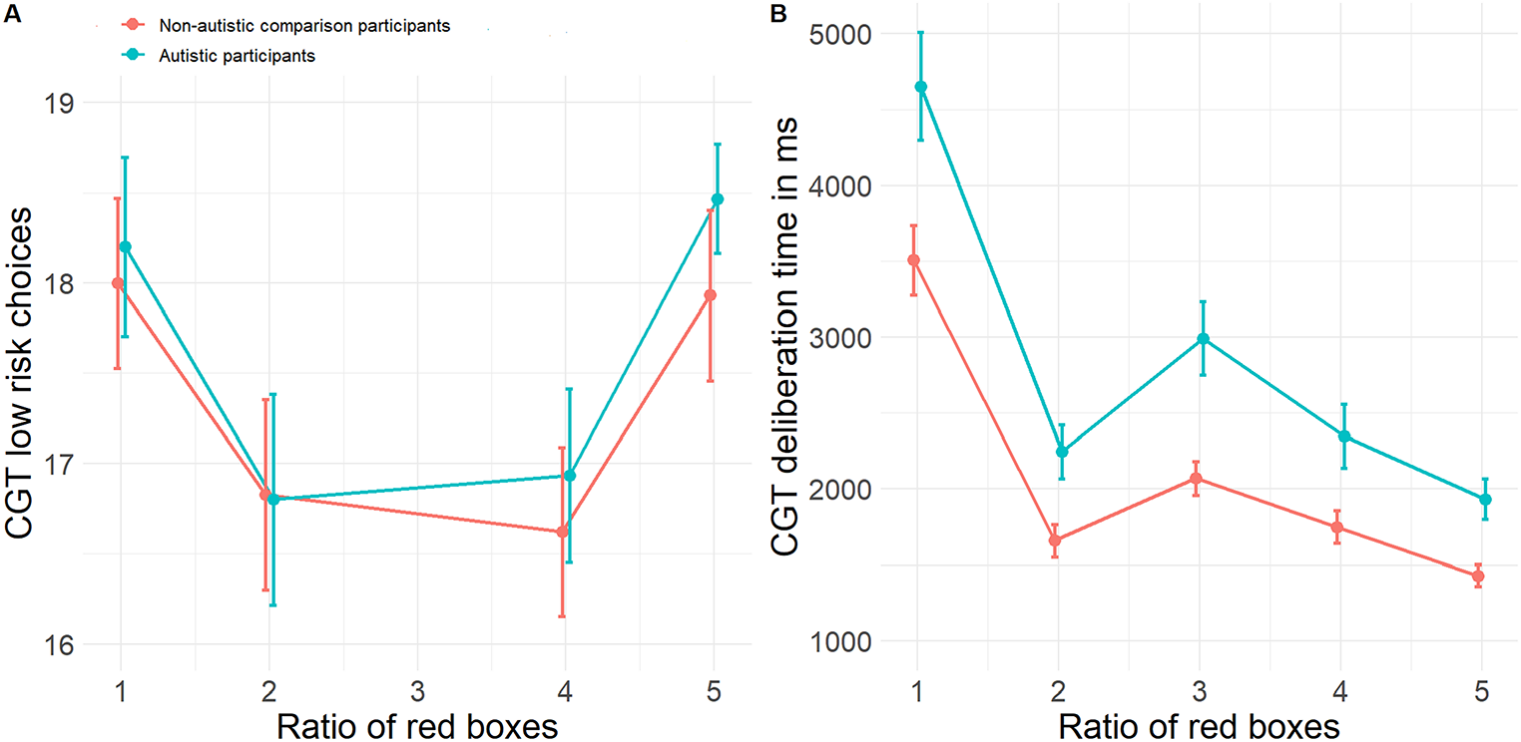
Low risk choices (**A**) and deliberation time (**B**) of the CRT *Note* Note that the equal risk ratio is not meaningful for the number of low risk choices. Error bars indicate standard errors

The effect of group was statistically significant (*b* = 1078.56, 95% CI [432.84, 1724.28], *t*(55) = 3.27, *p* = .001, β = 0.50, 95% CI [0.19, 0.80]), indicating that AP needed more time to make a decision, with a large effect size indicating a substantial impact. Moreover, we found a significant effect of risk ratio (*b* = -406.55, 95% CI [-521.89, -291.21], *t*(234) = -6.91, *p* < .001, β = − 0.41, 95% CI [-0.53, − 0.29]), showing that less time was needed for a decision when the risk ratio was higher. Sex and age did not significantly contribute to the model (Fig. 3; Supplement Table S6, S16-S17). When considering AP with and without comorbid depression, both required significantly more time to make decisions compared to NAP (see Supplement Table S18). However, upon integrating the BDI-II score into the analysis, we observed no significant group effect (see Supplement Table S19).

#### Low Risk Choices

None of the main or interaction effects of the model were statistically significant (Fig. 3; Supplement Table S20-S21).

### Hormones and Decision-Making Behavior

Basal cortisol, estradiol, and testosterone concentrations did not significantly distinguish between NAP and AP (see Table 2, and Supplement Table S22). When comparing hormonal levels of individuals of the same sex, we did not find significant group related differences either (see Table 2 and Supplement Table S19). In NAP, the regression model including age, sex, cortisol, and estradiol concentrations explained 51.0% of variance of the overall bet proportion of the CRT (*F*(4,24) = 6.24, *p* = .001; Table 3). Estradiol and cortisol levels were identified as significant predictors of CRT scores (transformed estradiol: [-0.02; 5.23]: β = 2.68, *p* = .027; CORT [0.02; 0.18]: β = 0.09, *p* = .025), while age ([-0.31; 0.06]: β = − 0.11, *p* = .263) and sex ([-9.39; 0.42]: β = -4.88, *p* = .062) were not. In AP, no hormone of interest was found to predict the CRT score. For the IGT scores, neither in NAP nor in AP basal hormone concentrations were identified as significant predictors (Supplement Tables S23-S26).

**Table 2:** Hormone levels of interest in the CRT subsample

	NAP		AP				NAP		AP				NAP		AP			
	Total						Females						Males					
	*N*		*N*				*N*		*n*				*n*		*n*			
**Participants**	**29**		**25**				**18**		**11**				**11**		**14**			
	*M*	*SD*	*M*	*SD*	*t/U*	*P*	*M*	*SD*	*M*	*SD*	***t/U***	*p*	*M*	*SD*	*M*	*SD*	*t/U*	*p*
Age in years	30.21	10.76	36.36	10.02	226.5^a^	0.018*	31.50	12.72	39.82	11.25	58.0^a^	0.065	28.09	6.44	33.64	8.36	-1.88	0.074
C in ng/ml	134.03	31.27	132.14	45.79	0.18	0.859	121.99	28.05	121.46	41.22	0.04	0.967	153.73	26.69	140.53	48.90	0.80	0.430
E in pg/ml	68.23	41.97	62.89	37.31	342.0^a^	0.722	82.10	47.64	79.18	51.17	96.0^a^	0.893	45.53	12.75	50.09	12.46	− 0.90	0.380
T in ng/ml	1.86	1.85	2.54	2.07	295.0^a^	0.242	0.46	0.16	0.42	0.21	0.54	0.596	4.14	0.55	4.20	1.04	− 0.17	0.866

*Note* CRT: Cambridge Risk Task; NAP: Non-autistic comparison participants; AP: Autistic participants; C: Cortisol; E: Estradiol; T: Testosterone

^a^ No normal distribution. Significance of parametric and non-parametric methods were not consistent. Results are based on Mann-Whitney-U-test

* Indicates *p* < .05; ** indicates *p* < .01

**Table 3:** Two-step hierarchical regression models for CRT scores

Groups	Steps	Predictors	*R* ^2^ (%)	∆ *R*^2^ (%)	F Change	Sig. F Change
NAP	1	Sex, Age	25.9	25.9	4.534	0.020*
	2	Sex, Age, cortisol, transformed estradiol	51.0	25.1	6.150	0.007**
AP	1	Sex, Age	5.3	5.3	0.610	0.552
	2	Sex, Age, cortisol, transformed estradiol	12.5	7.2	0.823	0.454

*Note* CRT: Cambridge Risk Task; NAP: Non-autistic comparison participants; AP: Autistic participants

Results are based on 1,000 bootstrap samples

*Indicates *p* < .05; **indicates *p* < .01

## Discussion

In the present study, we examined decision-making in autistic and non-autistic adults under ambiguity (i.e., as measured with the IGT, low outcome predictability, high risk) and under known risk conditions (i.e., as measured with the CRT, high outcome predictability, low risk). To elucidate possible underlying mechanisms, we examined the relationship of decision-making behavior with endocrine parameters that are thought to contribute to efficient decision-making.

In accordance with our hypothesis, decision-making under known risk conditions (low ambiguity) did not show any group specific differences regarding overall bet proportion or low risk choices. We could, therefore, not support previous conclusions that risk aversion per se (irrespective of the risk conditions) is a core feature of autism (De Martino et al., 2008; Gosling & Moutier, 2018). In line with our hypothesis that autism is associated with impaired decision-making under high ambiguity, we found worse performance of AP in later blocks of the IGT after excluding one influential case (defined by cook’s distance). When including comorbid depression in our analysis, we observed that only AP without comorbid depression displayed significantly poorer performance in later blocks of the IGT. Moreover, AP used a different strategy than their non-autistic counterparts and played `risk averse` by choosing more decks with predictable loss structure and frequent gains (deck B), while avoiding unpredictable losses with infrequent gains (deck A) in later blocks. This indicates that AP rather focused on the predictability of a choice option than on the reward outcome itself. Furthermore, AP needed more time to decide in the CRT, which is in line with results reported by Vella et al. (2018). Notably, we found slowed decision-making in AP only when not controlling for depressive symptoms (BDI-II). While basal estradiol and cortisol concentrations were associated with higher risk-taking in the CRT in NAP, hormonal levels were not associated with decision-making in AP.

Previous studies investigating decision-making in autistic individuals mostly evaluated performance on the IGT in young, usually male, participants with inconsistent findings. While a large meta-analysis found no difference in IGT performance between AP and NAP (Zeif & Yechiam, 2020), South and colleagues (2014) noticed that AP performed better in the IGT than NAP by avoiding potential losses rather than focusing on win chances. In contrast to these findings, but in line with our results, despite different inclusion and exclusion criteria, are two studies which showed impaired IGT performance of AP in later blocks (Mussey et al., 2015; Zhang et al., 2015). One of these studies (Mussey et al., 2015) related worse performance to frequent and fast switching between decks that negatively influenced learning effects. A narrative review of decision-making in autism evaluated different types of decision-making in AP and NAP. The authors suggest that AP and NAP perform similarly well at perceptual and reward-learning decisions, while they decide differently on metacognition and value-based paradigms (van der Plas et al., 2023). We think that this might explain highly variable study results in decision-making performance of AP. It has been argued that feeling-based signals are also strongly used to update decision-making in the IGT (Damasio, 1996) and intuitive decision-making processes cause problems in autistic participants. Contrary, a bias toward more logically decision-making was found (Brosnan et al., 2016; Vella et al., 2018). Thus, it might be that autistic individuals who used a value-based, intuitive, and introspective choice strategy, rather than a reward-based, cognitive strategy, perform worse in the IGT. While positive emotions tend to increase intuition based decisions, negative emotions activate executive control and, thus, provoke cognitive strategies (Schiebener & Brand, 2015). Therefore, autistic individuals in euthymic mood might perform worse than those with negative mood in later blocks of the IGT. Our discovery, that only AP without comorbid depression exhibit poorer performance in the later blocks of the IGT, aligns with this notion. This further highlights that depression might be associated with a reflective decision-making style. Moreover, individuals with strong executive control might avoid emotion-based decisions, and favor safer options (Schiebener & Brand, 2015). While our findings did not reveal impaired executive performance in autistic individuals, other scholars emphasize the significance of executive dysfunction associated with depression (Albantakis et al., 2020; Wallace et al., 2016). Johnston and colleagues (2019) investigated executive functions in autistic adults and found that two thirds were impaired in executive functions, potentially limiting the ability to reflect emotion-based risk-taking. However, it is important to note that the majority of our AP with comorbid depression took antidepressants, which can improve concentration and attention by reducing worrying and rumination for example. Thus, more studies should assess the participants’ affective state, evaluate individual decision-making styles, and incorporate a more comprehensive assessment of executive functions.

To evaluate the decision-making strategy, the frequency of gains and losses of chosen card decks might be more informative than the IGT net score. In our study, AP differed from NAP in the selection of “bad decks” by preferring decisions with predictable outcomes and frequent gains (deck B; losses from $1250) over those with unpredictable outcomes and infrequent gains (deck A; losses from $150–350$). Lin and colleagues (2007) described the preference for the disadvantageous final-outcome deck B over other decks as `prominent deck B phenomena` in NAP. Considering these phenomena, our study results imply that AP ’outperformed` the NAP. If the preference for card decks with frequent gains/infrequent losses in the IGT is regarded as more rationally driven behavior, our findings support the hypothesis of enhanced rationality as key feature of decision-making in autism. Contrary to our results, autistic individuals preferred deck A over the course of the game in the study by Zhang et al. (2015). These different outcomes might be related to divergent study designs including different characteristics of study participants (e.g., comorbidities). This might be critical, given certain limitations of the IGT, for example the susceptibility to person- and context-dependent variables (Schiebener & Brand, 2015). Context-dependent variables, such as the test environment and the presence of others, are likely to influence IGT performance. Brevers and colleagues (2015) manipulated context-dependent variables by examining IGT performance under various casino conditions, which included sound and red light in eighty non-gambler participants. They discovered that these conditions reduced the time taken for reflection and contemplation before responding to losses.

Although some studies suggest the presence of sex differences in decision-making, our study, along with numerous other laboratory investigations, found no discernible influence of sex on decision-making performance (Schiebener & Brand, 2015). Additionally, evidence suggests that cognitive abilities are not inherently sex-specific in the general population; rather, variations arise from environmental, cultural, practical, and hormonal factors (Jäncke, 2018). Moreover, even if sex differences were presumed to exist in IGT performance, they might not manifest in autism, as research indicates that sex differences observed in the general population are diminished or absent in individuals with ASD (Baron-Cohen et al., 2014).

Overall, we assume that autistic individuals do not generally exhibit decision-making deficits but avoid insecure outcomes under ambiguity as a manifestation of impaired prediction skills. An enhanced sensitivity to the feedback of losses/gains might relate to the choice of the lowest possible risk (low frequency loss) under unknown outcome conditions. This ‘risk averse’ decision-making pattern might also be relevant when socially engaging with others. For instance, autistic individuals tend to avoid new or unfamiliar situations, including encounters with new people and/or meeting them at new places. This aversion to ‘new things’, which might carry unknown risks, impedes autistic people in their everyday life and especially in social interactions with others. Thus, it would be interesting for future studies to explore whether the same decision-making patterns are observed in social contexts.

The complexity of decision-making is further complicated through the potential involvement of the hypothalamic-pituitary-adrenal and -gonadal axes. Elevated concentrations of androstendione, a precursor of testosterone, have previously been reported for autistic adults (Ruta et al., 2011; Schwarz et al., 2011). Although we did not measure androstendione in our study, we found no alteration in testosterone and estradiol levels in AP in comparison to NAP, which is line with observations by Ruta and colleagues (2011). Furthermore, we found basal cortisol and estradiol concentrations to predict decision-making under risk with known outcome probabilities, measured by the overall bet proportion in the CRT, in NAP but not AP. The absence of any hormonal predictive effect on decision-making behavior in AP might be explained by the heterogeneous character of our autistic sample. As mentioned before, in contrast to other studies, we included autistic individuals with psychiatric comorbidities and psychopharmaceutical medication, reflecting a typical clinical sample, which might lead to a broader variance in variables and results of the models. Thus, it is more challenging to identify single prediction factors. However, our findings in the non-autistic group are in line with results by other studies, which observed increased risk behavior when cortisol (Buckert et al., 2014) or estradiol (Kurath & Mata, 2018) levels were elevated. Furthermore, testosterone concentrations were neither associated with the CRT nor the IGT scores in either group. These results add to recent research indicating that testosterone is not as relevant for risk behavior as previously assumed (Derntl et., 2014).

### Strengths and Limitations

Our study contributes to a more detailed picture of decision-making and cognitive functions and a deeper understanding of the neurobiological character of these processes in AP. We achieved this by including male and female autistic adults, by assessing both decision-making under known probabilities and under ambiguity, and by combining neuropsychological and endocrine (sex and stress hormones) assessments. We moreover carefully controlled for depression that often co-occurs with autism and can be associated with decision-making difficulties (Albantakis et al., 2020; American Psychiatric Association, 2013; Hollocks et al., 2019). Finally, we considered influential cases and used two different analyses (net score and frequency-based) to evaluate decision-making processes in the IGT.

Despite these strengths, there are also limitations, which need to be addressed. First, NAP and AP were not perfectly matched regarding age and sex distribution, which is a common challenge in clinical trials with naturalistic designs (Fogel, 2018; Malay & Chung, 2012). To overcome this issue, sex and age were considered as covariates in the linear mixed models, and both variables were entered first to control for potential effects on risk behavior in hormonal regression models. Moreover, in our study, both AP and NAP had total IGT net scores below zero which can be considered as ‘low’ in comparison to a large student sample (Barnhart & Buelow, 2022) and challenges the validity of the reference level of our study. Second, half of the autistic group received different types of psychopharmacological treatment, which could have diverse effects on decision-making and hormonal levels (Moyer et al., 2019) or cognitive functions in general. For instance, antidepressants seem to exert a positive effect on cognitive functions in depressive and anxiety disorders (Amado-Boccara, 1995; Krysta et al., 2015). Third, decision-making tasks used in this study did not include everyday life scenarios, which might be more informative for potential therapeutic interventions in AP. Finally, we did not include emotional measures that might also be relevant for non-social decision-making processes. Thus, future studies should also include social decision-making paradigms to further tease apart the contributions of cognitive and emotional factors in larger samples with/without comorbid depression.

## Conclusion

Our study results suggest that AP are not generally impaired in decision-making; however, they may adopt a different decision-making approach compared to NAP. AP used a ’risk-averse’ strategy of decision-making under ambiguity, which was characterized by an avoidance of insecure and unpredictable losses during the IGT. On the contrary, we found similar performance in decision-making under risk when outcome probabilities were known (CRT). AP also needed more time to decide. Subanalyses indicated that individuals with AP, but without comorbid depression, performed worse on tasks measuring decision-making under ambiguity. This suggests a potential link between depression and more reflective decision-making, which may be advantageous in this context.

Our findings are therefore consistent with the notion that main characteristics of AP might manifest as a function of underlying compromised prediction skills. Thus, we recommend being as transparent and precise as possible when interacting with autistic individuals. This will provide a secure framework, which is of particular importance for single or group based psychotherapy with autistic patients (Schilbach et al., 2022). Future research should investigate the mechanisms underlying predictive uncertainties in decision-making behavior, specifically the risk averse decision-making in social situations, by considering person dependent variables such as mood and individual decision-making styles.

## Electronic supplementary material

Below is the link to the electronic supplementary material.

Supplementary Material 1

## Data Availability

The data or materials for the experiments reported here is available on reasonable request from the corresponding author, the study was not preregistered.
