# Supplementary material for: Autistic Adults Avoid Unpredictability in Decision-Making
Source: J Autism Dev Disord. 2024 Aug 19;55(12):4234–46. doi: 10.1007/s10803-024-06503-2 (PMC12589206; doi:10.1007/s10803-024-06503-2)
Supplement: Supplementary file 1 — Supplementary Material 1 [file 10803_2024_6503_MOESM1_ESM.docx]

**Supplementary material**

**Autistic adults avoid unpredictability in decision-making.**

**Journal of Autism and Developmental Disorders**

Ana Macchia^1,2*^, Laura Albantakis^3,4, 5*^, Paul Theo Zebhauser^6^, Marie-Luise Brandi^3^, Leonhard Schilbach^3,4,7,8^, Anna-Katharine Brem^1,9,10^

**^1^** Max Planck Institute of Psychiatry, Munich, Germany

**^2^** Clinic for Psychiatry/Psychotherapy III, Ulm University, Ulm, Germany

**^3^** Independent Max Planck Research Group for Social Neuroscience, Max Planck Institute of Psychiatry, Munich, Germany

**^4^** International Max Planck Research School for Translational Psychiatry, Munich, Germany

^5^ Department of Psychiatry and Psychotherapy, LMU University Hospital, LMU Munich, Germany

**^6^**Technical University of Munich, Germany; School of Medicine, Department of Neurology

**^7^** LVR-Klinikum Duesseldorf/Kliniken der Heinrich-Heine-Universitaet, Department of General Psychiatry 2, Duesseldorf, Germany

**^8^** Ludwig-Maximilian-University, Munich, Germany

^9^University Hospital of Old Age Psychiatry, University of Bern, Bern, Switzerland

^10^Department of Old Age Psychiatry, Institute of Psychiatry, Psychology and Neuroscience, King’s College London, London, United Kingdom

* The authors contributed equally to this work (shared first authorship)

Corresponding author: Anna-Katharine Brem, King's College London, UK. Email: katy.brem@gmail.com. Tel. +41793641999.

| **Table S1**  *Medication intake of AP (N = 32)* | | |
| --- | --- | --- |
|  | *n* | % |
| Antidepressants | 8 | 25% |
| Antipsychotics | 2 | 6.25% |
| Stimulants | 1 | 3.13% |
| Benzodiazepines | 1 | 3.13% |
| Combination* | 4 | 12.5% |
| *Note.* AP: Autistic participants. *Three times combination of antidepressants and antipsychotics. One time combination of antidepressants and stimulants. | | |

| **Table S2**  *Test of Normality (Shapiro-Wilk) for NAP.* | | |
| --- | --- | --- |
| Variable | *W* | *p* |
| age | 0.855 | .001** |
| BDI II - Score | 0.828 | <.001** |
| IGT Score | 0.965 | .413 |
| CGT overall bet proportion | 0.913 | .021* |
| TMT A | 0.891 | .004** |
| TMT B | 0.762 | <.001** |
| TMT B/A | 0.856 | <.001** |
| *Note.* NAP: Non-autistic comparison participants. Significant results suggest a deviation from normality.  * Indicates p < .05. ** indicates p < .01. | | |

| **Table S3**  *Test of Normality (Shapiro-Wilk) for AP.* | | |
| --- | --- | --- |
| Variable | *W* | *p* |
| age | 0.939 | .072 |
| BDI II - Score | 0.904 | .008** |
| IGT Score | 0.950 | .144 |
| CGT overall bet proportion | 0.880 | .003** |
| TMT-A | 0.883 | .002** |
| TMT-B | 0.901 | .007** |
| TMT-B/A | 0.960 | 0.280 |
| *Note.*  Autistic participants. Significant results suggest a deviation from normality.  * Indicates p < .05. ** indicates p < .01. | | |

| **Table S4**  *Descriptive statistics for the IGT net score in each 20-trial block* | | | | | |
| --- | --- | --- | --- | --- | --- |
|  | **NAP**  **(*N* = 30)** | |  | **AP**  **(*N* = 32)** | |
|  | *M* | *SD* |  | *M* | *SD* |
| Block 1 | -4.93 | 6.84 |  | -1.69 | 6.03 |
| Block 2 | -2.07 | 7.58 |  | -1.25 | 6.75 |
| Block 3 | 0.27 | 7.46 |  | -1.25 | 8.44 |
| Block 4 | 0.00 | 7.74 |  | 0.44 | 10.75 |
| Block 5 | 0.60 | 8.12 |  | 0.12 | 10.91 |
| Total net Score | -6.13 | 23.02 |  | -3.62 | 35.15 |
| *Note.* Descriptive statistics including one influential case (*N*=62). IGT: Iowa Gambling Task; NAP: Non-autistic comparison participants; AP: Autistic participants; Total net Score: advantageous – disadvantageous card decks. | | | | | |

| **Table S5**  *Descriptive statistics for the IGT net score in each 20-trial block* | | | | | |
| --- | --- | --- | --- | --- | --- |
|  | **NAP**  **(*N* = 30)** | |  | **AP**  **(*N* = 31)** | |
|  | *M* | *SD* |  | *M* | *SD* |
| Block 1 | -4.93 | 6.84 |  | -1.68 | 6.13 |
| Block 2 | -2.07 | 7.58 |  | -0.09 | 6.57 |
| Block 3 | 0.27 | 7.46 |  | -1.94 | 7.62 |
| Block 4 | 0.00 | 7.74 |  | -0.19 | 10.31 |
| Block 5 | 0.60 | 8.12 |  | -0.52 | 10.46 |
| Total net Score | -6.13 | 23.02 |  | -3.62 | 35.15 |
| *Note.* Descriptive statistics excluding one influential case (*N*=61). IGT: Iowa Gambling Task; NAP: Non-autistic comparison participants; AP: Autistic participants; Total net Score: advantageous – disadvantageous card decks. | | | | | |

| **Table S6**  *Descriptive statistics for the CRT.* | | | | | |
| --- | --- | --- | --- | --- | --- |
|  | **NAP (*N* = 29)** | | **AP (*N* = 30)** | | |
|  | *M* | *SD* | | *M* | *SD* |
| **Overall bet proportion** | 544.56 | 279.72 | | 515.57 | 273.36 |
| **Deliberation time [ms]** | 2,083.00 | 523.95 | | 2,834.39 | 959.53 |
| Ratio 1/6 | 3,507.63 | 1,244.14 | | 4,651.12 | 1,941.84 |
| Ratio 2/6 | 1,658.68 | 584.12 | | 2,246.09 | 977.53 |
| Ratio 3/6 | 2,069.96 | 596.48 | | 2,993.23 | 1,325.23 |
| Ratio 4/6 | 1,748.94 | 575.89 | | 2,347.62 | 1,151.17 |
| Ratio 5/6 | 1,429.76 | 398.83 | | 1,933.91 | 740.46 |
| **Low risk choices** | 69.38 | 9.06 | | 70.40 | 8.61 |
| Ratio 1/6 | 18.00 | 2.54 | | 18.20 | 2.71 |
| Ratio 2/6 | 16.83 | 2.84 | | 16.80 | 3.20 |
| Ratio 4/6 | 16.62 | 2.51 | | 16.93 | 2.64 |
| Ratio 5/6 | 17.93 | 2.55 | | 18.47 | 1.66 |
| *Note.* CRT: Cambridge risk task; NAP: Non-autistic comparison participants; AP: Autistic participants; overall bet proportion: Mean score obtained across all trials); deliberation time: mean choice reaction time; Low risk choices: Number of choices of the more probable option. | | | | | |

| **Table S7** |  | | | | | | |
| --- | --- | --- | --- | --- | --- | --- | --- |
|  | **IGT Score** | | | | | | |
| *Predictors* | *Estimates* | *std. Beta* | *CI* | *standardized CI* | *p* | *std. p* | *df* |
| (Intercept) | -6.35 | -0.17 | -10.05 – -2.66 | -0.54 – 0.19 | **0.001** | 0.351 | 58.00 |
| group [1] | 3.72 | 0.17 | -0.65 – 8.09 | -0.22 – 0.55 | 0.097 | 0.404 | 58.00 |
| block | 1.31 | 0.23 | 0.60 – 2.02 | 0.10 – 0.35 | **<0.001** | **<0.001** | 246.00 |
| age | -1.08 | -0.13 | -2.67 – 0.51 | -0.32 – 0.06 | 0.188 | 0.188 | 58.00 |
| sex [1] | 1.38 | 0.17 | -1.74 – 4.50 | -0.21 – 0.55 | 0.388 | 0.388 | 58.00 |
| group [1] * block | -0.78 | -0.13 | -1.77 – 0.21 | -0.30 – 0.04 | 0.123 | 0.123 | 246.00 |
| **Random Effects** | | | | | | | |
| σ^2^ | 39.53 | | | | | | |
| τ_00_ _subject_ | 27.81 | | | | | | |
| ICC | 0.41 | | | | | | |
| N _subject_ | 62 | | | | | | |
| Observations | 310 | | | | | | |
| Marginal R^2^ / Conditional R^2^ | 0.046 / 0.440 | | | | | | |

| **Table S8** |  | | | | | | |
| --- | --- | --- | --- | --- | --- | --- | --- |
|  | **IGT Score (without influential case)** | | | | | | |
| *Predictors* | *Estimates* | *std. Beta* | *CI* | *standardized CI* | *p* | *std. p* | *df* |
| (Intercept) | -6.03 | -0.12 | -9.68 – -2.37 | -0.50 – 0.26 | **0.002** | 0.536 | 57.00 |
| group [1] | 3.93 | 0.11 | -0.40 – 8.27 | -0.29 – 0.52 | 0.077 | 0.587 | 57.00 |
| block | 1.31 | 0.23 | 0.63 – 2.00 | 0.11 – 0.35 | **<0.001** | **<0.001** | 242.00 |
| age | -0.88 | -0.11 | -2.48 – 0.73 | -0.31 – 0.09 | 0.289 | 0.289 | 57.00 |
| sex [1] | 0.96 | 0.12 | -2.19 – 4.11 | -0.27 – 0.51 | 0.552 | 0.552 | 57.00 |
| group [1] * block | -1.01 | -0.18 | -1.97 – -0.05 | -0.35 – -0.01 | **0.040** | **0.040** | 242.00 |
| **Random Effects** | | | | | | | |
| σ^2^ | 36.61 | | | | | | |
| τ_00_ _subject_ | 27.78 | | | | | | |
| ICC | 0.43 | | | | | | |
| N _subject_ | 61 | | | | | | |
| Observations | 305 | | | | | | |
| Marginal R^2^ / Conditional R^2^ | 0.038 / 0.453 | | | | | | |

| **Table S9** | **IGT Score for participants with and without Depression** | | | | | | |
| --- | --- | --- | --- | --- | --- | --- | --- |
| *Predictors* | *Estimates* | *std. Beta* | *CI* | *standardized CI* | *p* | *std. p* | *df* |
| (Intercept) | -6.37 | -0.18 | -9.96 – -2.77 | -0.53 – 0.18 | **0.001** | 0.322 | 57.00 |
| AP no Depression [1] | 3.38 | -0.17 | -1.83 – 8.58 | -0.63 – 0.28 | 0.202 | 0.441 | 57.00 |
| AP Depression [2] | 4.26 | 0.50 | -0.82 – 9.33 | 0.05 – 0.94 | 0.100 | **0.030** | 57.00 |
| block | 1.31 | 0.23 | 0.60 – 2.02 | 0.10 – 0.35 | **<0.001** | **<0.001** | 245.00 |
| age | -1.34 | -0.16 | -2.89 – 0.21 | -0.35 – 0.03 | 0.090 | 0.090 | 57.00 |
| sex [1] | 1.29 | 0.16 | -1.74 – 4.31 | -0.21 – 0.52 | 0.398 | 0.398 | 57.00 |
| AP no Depression [1] × block | -1.61 | -0.28 | -2.84 – -0.38 | -0.49 – -0.06 | **0.011** | **0.011** | 245.00 |
| AP Depression [2] × block | -0.05 | -0.01 | -1.23 – 1.12 | -0.21 – 0.19 | 0.928 | 0.928 | 245.00 |
| **Random Effects** | | | | | | | |
| σ^2^ | 38.90 | | | | | | |
| τ_00_ _subject_ | 24.35 | | | | | | |
| ICC | 0.38 | | | | | | |
| N _subject_ | 62 | | | | | | |
| Observations | 310 | | | | | | |
| Marginal R^2^ / Conditional R^2^ | 0.109 / 0.452 | | | | | | |

| **Table S10** | **IGT Score including BDI-II** | | | | | | |
| --- | --- | --- | --- | --- | --- | --- | --- |
| *Predictors* | *Estimates* | *std. Beta* | *CI* | *standardized CI* | *p* | *std. p* | *df* |
| (Intercept) | -7.18 | -0.28 | -11.29 – -3.07 | -0.70 – 0.15 | **0.001** | 0.202 | 57.00 |
| group [1] | 4.66 | 0.28 | -0.15 – 9.47 | -0.18 – 0.74 | 0.058 | 0.230 | 57.00 |
| block | 1.31 | 0.23 | 0.60 – 2.03 | 0.10 – 0.35 | **<0.001** | **<0.001** | 246.00 |
| BDI | -0.89 | -0.11 | -2.75 – 0.97 | -0.33 – 0.12 | 0.341 | 0.341 | 57.00 |
| age | -1.10 | -0.13 | -2.73 – 0.52 | -0.33 – 0.06 | 0.180 | 0.180 | 57.00 |
| sex [1] | 2.04 | 0.25 | -1.43 – 5.51 | -0.17 – 0.67 | 0.244 | 0.244 | 57.00 |
| sex [1] × block | -0.78 | -0.13 | -1.78 – 0.21 | -0.30 – 0.04 | 0.123 | 0.123 | 246.00 |
| **Random Effects** | | | | | | | |
| σ^2^ | 39.53 | | | | | | |
| τ_00_ _subject_ | 27.86 | | | | | | |
| ICC | 0.41 | | | | | | |
| N _subject_ | 62 | | | | | | |
| Observations | 310 | | | | | | |
| Marginal R^2^ / Conditional R^2^ | 0.053 / 0.445 | | | | | | |

| **Table S11** |  | | | | | | |
| --- | --- | --- | --- | --- | --- | --- | --- |
|  | **Deck A** | | | | | | |
| *Predictors* | *Estimates* | *std. Beta* | *CI* | *standardized CI* | *p* | *std. p* | *df* |
| (Intercept) | 3.59 | 0.17 | 2.69 – 4.50 | -0.15 – 0.49 | **<0.001** | 0.298 | 58.00 |
| group [1] | 0.48 | -0.21 | -0.63 – 1.58 | -0.55 – 0.13 | 0.397 | 0.224 | 58.00 |
| block | -0.14 | -0.09 | -0.34 – 0.06 | -0.23 – 0.04 | 0.172 | 0.172 | 246.00 |
| age | 0.06 | 0.03 | -0.30 – 0.42 | -0.14 – 0.20 | 0.731 | 0.731 | 58.00 |
| sex [1] | -0.25 | -0.12 | -0.95 – 0.46 | -0.45 – 0.22 | 0.496 | 0.496 | 58.00 |
| group [1] * block | -0.31 | -0.21 | -0.59 – -0.03 | -0.39 – -0.02 | **0.030** | **0.030** | 246.00 |
| **Random Effects** | | | | | | | |
| σ^2^ | 3.14 | | | | | | |
| τ_00_ _subject_ | 1.18 | | | | | | |
| ICC | 0.27 | | | | | | |
| N _subject_ | 62 | | | | | | |
| Observations | 310 | | | | | | |
| Marginal R^2^ / Conditional R^2^ | 0.060 / 0.318 | | | | | | |

| **Table S12** |  | | | | | | |
| --- | --- | --- | --- | --- | --- | --- | --- |
|  | **Deck B** | | | | | | |
| *Predictors* | *Estimates* | *std. Beta* | *CI* | *standardized CI* | *p* | *std. p* | *df* |
| (Intercept) | 9.58 | 0.09 | 7.71 – 11.45 | -0.29 – 0.46 | **<0.001** | 0.651 | 58.00 |
| group [1] | -2.34 | -0.06 | -4.54 – -0.13 | -0.45 – 0.34 | **0.039** | 0.780 | 58.00 |
| block | -0.52 | -0.18 | -0.87 – -0.16 | -0.30 – -0.06 | **0.004** | **0.004** | 246.00 |
| age | 0.48 | 0.12 | -0.34 – 1.29 | -0.08 – 0.31 | 0.255 | 0.255 | 58.00 |
| sex [1] | -0.45 | -0.11 | -2.04 – 1.15 | -0.50 – 0.28 | 0.587 | 0.587 | 58.00 |
| group [1] * block | 0.70 | 0.24 | 0.21 – 1.19 | 0.07 – 0.41 | **0.005** | **0.005** | 246.00 |
| **Random Effects** | | | | | | | |
| σ^2^ | 9.67 | | | | | | |
| τ_00_ _subject_ | 7.45 | | | | | | |
| ICC | 0.43 | | | | | | |
| N _subject_ | 62 | | | | | | |
| Observations | 310 | | | | | | |
| Marginal R^2^ / Conditional R^2^ | 0.029 / 0.451 | | | | | | |

| **Table S13** |  | | | | | | |
| --- | --- | --- | --- | --- | --- | --- | --- |
|  | **Deck C** | | | | | | |
| *Predictors* | *Estimates* | *std. Beta* | *CI* | *standardized CI* | *p* | *std. p* | *df* |
| (Intercept) | 3.30 | -0.01 | 1.98 – 4.62 | -0.36 – 0.33 | **<0.001** | 0.939 | 58.00 |
| group [1] | 0.29 | 0.01 | -1.31 – 1.89 | -0.35 – 0.37 | 0.720 | 0.962 | 58.00 |
| block | 0.02 | 0.01 | -0.26 – 0.31 | -0.12 – 0.15 | 0.872 | 0.872 | 246.00 |
| age | -0.04 | -0.01 | -0.58 – 0.49 | -0.19 – 0.17 | 0.877 | 0.877 | 58.00 |
| sex [1] | 0.05 | 0.02 | -1.00 – 1.10 | -0.34 – 0.37 | 0.926 | 0.926 | 58.00 |
| group [1] * block | -0.09 | -0.04 | -0.48 – 0.31 | -0.23 – 0.15 | 0.659 | 0.659 | 246.00 |
| **Random Effects** | | | | | | | |
| σ^2^ | 6.28 | | | | | | |
| τ_00_ _subject_ | 2.77 | | | | | | |
| ICC | 0.31 | | | | | | |
| N _subject_ | 62 | | | | | | |
| Observations | 310 | | | | | | |
| Marginal R^2^ / Conditional R^2^ | 0.001 / 0.307 | | | | | | |

| **Table S14** |  | | | | | | |
| --- | --- | --- | --- | --- | --- | --- | --- |
|  | **Deck C (order norm transformation)** | | | | | | |
| *Predictors* | *Estimates* | *std. Beta* | *CI* | *standardized CI* | *p* | *std. p* | *df* |
| (Intercept) | -0.38 | -0.12 | -0.76 – 0.01 | -0.35 – 0.11 | 0.058 | 0.297 | 58.00 |
| group [1] | 0.45 | 0.15 | -0.05 – 0.95 | -0.09 – 0.39 | 0.081 | 0.233 | 58.00 |
| block | 0.09 | 0.14 | -0.01 – 0.20 | -0.02 – 0.30 | 0.091 | 0.091 | 246.00 |
| age | -0.06 | -0.07 | -0.18 – 0.05 | -0.19 – 0.05 | 0.284 | 0.284 | 58.00 |
| sex [1] | 0.08 | 0.09 | -0.14 – 0.30 | -0.15 – 0.32 | 0.481 | 0.481 | 58.00 |
| group [1] * block | -0.10 | -0.15 | -0.25 – 0.05 | -0.38 – 0.07 | 0.179 | 0.179 | 246.00 |
| **Random Effects** | | | | | | | |
| σ^2^ | 0.89 | | | | | | |
| τ_00_ _subject_ | 0.00 | | | | | | |
| ICC | 0.00 | | | | | | |
| N _subject_ | 62 | | | | | | |
| Observations | 310 | | | | | | |
| Marginal R^2^ / Conditional R^2^ | 0.016 / 0.019 | | | | | | |

| **Table S15** |  | | | | | | |
| --- | --- | --- | --- | --- | --- | --- | --- |
|  | **Deck D** | | | | | | |
| *Predictors* | *Estimates* | *std. Beta* | *CI* | *standardized CI* | *p* | *std. p* | *df* |
| (Intercept) | 3.52 | -0.17 | 1.66 – 5.39 | -0.54 – 0.21 | **<0.001** | 0.393 | 58.00 |
| group [1] | 1.57 | 0.16 | -0.62 – 3.76 | -0.24 – 0.56 | 0.163 | 0.436 | 58.00 |
| block | 0.63 | 0.22 | 0.29 – 0.98 | 0.10 – 0.34 | **<0.001** | **<0.001** | 246.00 |
| age | -0.50 | -0.12 | -1.32 – 0.32 | -0.32 – 0.08 | 0.238 | 0.238 | 58.00 |
| sex [1] | 0.64 | 0.16 | -0.97 – 2.25 | -0.23 – 0.54 | 0.437 | 0.437 | 58.00 |
| group [1] * block | -0.30 | -0.10 | -0.78 – 0.18 | -0.27 – 0.06 | 0.218 | 0.218 | 246.00 |
| **Random Effects** | | | | | | | |
| σ^2^ | 9.27 | | | | | | |
| τ_00_ _subject_ | 7.64 | | | | | | |
| ICC | 0.45 | | | | | | |
| N _subject_ | 62 | | | | | | |
| Observations | 310 | | | | | | |
| Marginal R^2^ / Conditional R^2^ | 0.044 / 0.476 | | | | | | |

**CGT overall bet proportion (root transformation):** The model was not statistically significant, R² = .05, R² adjusted = -.003, F(3, 55) = 0.93, p = .431. There was no significant influence of group (β = -.14, p = .615, 95%CI [-.70, .42]), sex (β = -.30, p = .284, 95%CI [-.84, .25]) or age (β = -.12, p = .416, 95%CI [-.40, .17]) on the overall bed proportion of the CGT.

| **Table S16** |  | | | | | | |
| --- | --- | --- | --- | --- | --- | --- | --- |
|  | **Deliberation time CGT** | | | | | | |
| *Predictors* | *Estimates* | *std. Beta* | *CI* | *standardized CI* | *p* | *std. p* | *df* |
| (Intercept) | 3333.57 | -0.25 | 2804.04 – 3863.09 | -0.53 – 0.04 | **<0.001** | 0.092 | 55.00 |
| group [1] | 1078.56 | 0.50 | 432.84 – 1724.28 | 0.19 – 0.80 | **0.001** | **0.002** | 55.00 |
| risk ratio | -406.55 | -0.41 | -521.89 – -291.21 | -0.53 – -0.29 | **<0.001** | **<0.001** | 234.00 |
| age | 97.58 | 0.07 | -117.44 – 312.59 | -0.08 – 0.22 | 0.378 | 0.378 | 55.00 |
| sex [1] | -7.27 | -0.01 | -427.59 – 413.04 | -0.30 – 0.29 | 0.973 | 0.973 | 55.00 |
| group [1] * risk ratio | -126.74 | -0.13 | -288.50 – 35.02 | -0.29 – 0.04 | 0.126 | 0.126 | 234.00 |
| **Random Effects** | | | | | | | |
| σ^2^ | 1004365.44 | | | | | | |
| τ_00_ _subject_ | 415050.94 | | | | | | |
| ICC | 0.29 | | | | | | |
| N _subject_ | 59 | | | | | | |
| Observations | 295 | | | | | | |
| Marginal R^2^ / Conditional R^2^ | 0.298 / 0.504 | | | | | | |

| **Table S17** |  | | | | | | |
| --- | --- | --- | --- | --- | --- | --- | --- |
|  | **Deliberation time CGT (order norm transformation)** | | | | | | |
| *Predictors* | *Estimates* | *std. Beta* | *CI* | *standardized CI* | *p* | *std. p* | *df* |
| (Intercept) | 0.78 | -0.16 | 0.38 – 1.18 | -0.49 – 0.18 | **<0.001** | 0.372 | 55.00 |
| group [1] | 0.45 | 0.45 | -0.02 – 0.93 | 0.09 – 0.80 | 0.060 | **0.019** | 55.00 |
| risk ratio | -0.31 | -0.44 | -0.38 – -0.24 | -0.54 – -0.34 | **<0.001** | **<0.001** | 234.00 |
| age | 0.10 | 0.10 | -0.08 – 0.28 | -0.08 – 0.28 | 0.277 | 0.277 | 55.00 |
| sex [1] | -0.13 | -0.13 | -0.49 – 0.22 | -0.49 – 0.22 | 0.473 | 0.473 | 55.00 |
| group [1] * risk ratio | -0.00 | -0.00 | -0.10 – 0.10 | -0.15 – 0.14 | 0.951 | 0.951 | 234.00 |
| **Random Effects** | | | | | | | |
| σ^2^ | 0.39 | | | | | | |
| τ_00_ _subject_ | 0.36 | | | | | | |
| ICC | 0.48 | | | | | | |
| N _subject_ | 59 | | | | | | |
| Observations | 295 | | | | | | |
| Marginal R^2^ / Conditional R^2^ | 0.268 / 0.618 | | | | | | |

| **Table S18** | **Deliberation time CGT for participants with and without Depression (square root transformation)** | | | | | | |
| --- | --- | --- | --- | --- | --- | --- | --- |
| *Predictors* | *Estimates* | *std. Beta* | *CI* | *standardized CI* | *p* | *std. p* | *df* |
| (Intercept) | 3333.42 | -0.25 | 2796.07 – 3870.76 | -0.54 – 0.04 | **<0.001** | 0.095 | 54.00 |
| AP no Depression [1] | 1098.06 | 0.49 | 294.89 – 1901.22 | 0.12 – 0.87 | **0.008** | **0.011** | 54.00 |
| AP Depression [2] | 1062.22 | 0.50 | 279.34 – 1845.09 | 0.12 – 0.88 | **0.008** | **0.010** | 54.00 |
| ratio red | -406.55 | -0.41 | -522.74 – -290.36 | -0.53 – -0.29 | **<0.001** | **<0.001** | 233.00 |
| age | 96.84 | 0.07 | -128.09 – 321.77 | -0.09 – 0.23 | 0.392 | 0.392 | 54.00 |
| sex[1] | -7.36 | -0.01 | -441.28 – 426.57 | -0.31 – 0.30 | 0.973 | 0.973 | 54.00 |
| AP no Depression [1] × ratio red | -135.24 | -0.14 | -338.86 – 68.39 | -0.34 – 0.07 | 0.192 | 0.192 | 233.00 |
| AP Depression [2] × ratio red | -119.31 | -0.12 | -314.16 – 75.55 | -0.32 – 0.08 | 0.229 | 0.229 | 233.00 |
| **Random Effects** | | | | | | | |
| σ^2^ | 1008594.70 | | | | | | |
| τ_00_ _subject_ | 425591.90 | | | | | | |
| ICC | 0.30 | | | | | | |
| N _subject_ | 59 | | | | | | |
| Observations | 295 | | | | | | |
| Marginal R^2^ / Conditional R^2^ | 0.296 / 0.505 | | | | | | |

| **Table S19** | **Deliberation time CGT including BDI-II (order norm transformation)** | | | | | | |
| --- | --- | --- | --- | --- | --- | --- | --- |
| *Predictors* | *Estimates* | *std. Beta* | *CI* | *standardized CI* | *p* | *std. p* | *df* |
| (Intercept) | 0.90 | -0.03 | 0.45 – 1.35 | -0.43 – 0.36 | **<0.001** | 0.868 | 54.00 |
| group [1] | 0.32 | 0.31 | -0.21 – 0.84 | -0.12 – 0.73 | 0.233 | 0.157 | 54.00 |
| ratio red | -0.31 | -0.44 | -0.38 – -0.24 | -0.55 – -0.34 | **<0.001** | **<0.001** | 234.00 |
| age centered | 0.11 | 0.11 | -0.08 – 0.29 | -0.08 – 0.29 | 0.254 | 0.254 | 54.00 |
| gender [1] | -0.23 | -0.23 | -0.62 – 0.17 | -0.62 – 0.17 | 0.252 | 0.252 | 54.00 |
| BDI centered | 0.13 | 0.13 | -0.08 – 0.35 | -0.08 – 0.34 | 0.212 | 0.212 | 54.00 |
| group [1] × ratio red | -0.00 | -0.00 | -0.10 – 0.10 | -0.15 – 0.14 | 0.951 | 0.951 | 234.00 |
| **Random Effects** | | | | | | | |
| σ^2^ | 0.39 | | | | | | |
| τ_00_ _subject_ | 0.36 | | | | | | |
| ICC | 0.47 | | | | | | |
| N _subject_ | 59 | | | | | | |
| Observations | 295 | | | | | | |
| Marginal R^2^ / Conditional R^2^ | 0.277 / 0.621 | | | | | | |

| **Table S20** |  | | | | | | |
| --- | --- | --- | --- | --- | --- | --- | --- |
|  | **Low risk choices CGT** | | | | | | |
| *Predictors* | *Estimates* | *std. Beta* | *CI* | *standardized CI* | *p* | *std. p* | *df* |
| (Intercept) | 17.61 | 0.01 | 16.31 – 18.91 | -0.42 – 0.44 | **<0.001** | 0.954 | 55.00 |
| group [1] | -0.17 | 0.05 | -1.66 – 1.33 | -0.40 – 0.51 | 0.829 | 0.824 | 55.00 |
| risk ratio | -0.03 | -0.02 | -0.24 – 0.17 | -0.14 – 0.10 | 0.742 | 0.742 | 175.00 |
| age | 0.16 | 0.06 | -0.45 – 0.78 | -0.17 – 0.29 | 0.604 | 0.604 | 55.00 |
| sex [1] | -0.19 | -0.07 | -1.39 – 1.01 | -0.52 – 0.38 | 0.754 | 0.754 | 55.00 |
| group [1] * risk ratio | 0.10 | 0.06 | -0.19 – 0.39 | -0.11 – 0.23 | 0.491 | 0.491 | 175.00 |
| **Random Effects** | | | | | | | |
| σ^2^ | 3.16 | | | | | | |
| τ_00_ _subject_ | 4.24 | | | | | | |
| ICC | 0.57 | | | | | | |
| N _subject_ | 59 | | | | | | |
| Observations | 236 | | | | | | |
| Marginal R^2^ / Conditional R^2^ | 0.007 / 0.575 | | | | | | |

| **Table S21** |  | | | | | | |
| --- | --- | --- | --- | --- | --- | --- | --- |
|  | **Low risk choices CGT (order norm transformation)** | | | | | | |
| *Predictors* | *Estimates* | *std. Beta* | *CI* | *standardized CI* | *p* | *std. p* | *df* |
| (Intercept) | 0.04 | 0.04 | -0.40 – 0.48 | -0.39 – 0.47 | 0.858 | 0.850 | 55.00 |
| group [1] | -0.02 | 0.05 | -0.52 – 0.49 | -0.41 – 0.50 | 0.946 | 0.847 | 55.00 |
| risk ratio | -0.01 | -0.02 | -0.08 – 0.06 | -0.14 – 0.10 | 0.717 | 0.717 | 175.00 |
| age | 0.07 | 0.08 | -0.14 – 0.28 | -0.15 – 0.31 | 0.507 | 0.507 | 55.00 |
| sex [1] | -0.11 | -0.12 | -0.52 – 0.30 | -0.57 – 0.33 | 0.605 | 0.605 | 55.00 |
| group [1] * risk ratio | 0.02 | 0.03 | -0.08 – 0.12 | -0.14 – 0.20 | 0.696 | 0.696 | 175.00 |
| **Random Effects** | | | | | | | |
| σ^2^ | 0.37 | | | | | | |
| τ_00_ _subject_ | 0.49 | | | | | | |
| ICC | 0.57 | | | | | | |
| N _subject_ | 59 | | | | | | |
| Observations | 236 | | | | | | |
| Marginal R^2^ / Conditional R^2^ | - 1. 0.574 | | | | | | |

| **Table S22** | | | | | | | | | | | | | | | | | | | |
| --- | --- | --- | --- | --- | --- | --- | --- | --- | --- | --- | --- | --- | --- | --- | --- | --- | --- | --- | --- |
| *Hormone levels of interest in the IGT subsample* | | | | | | | | | | | | | | | | | | | |
|  | **NAP** | | **AP** | |  | | **NAP** | | **AP** | |  | | **NAP** | | **AP** | |  | |  |
|  | Total | | | |  |  | Females | | | |  |  | Males | | | |  |  |  |
|  | *n* | | *n* | |  |  | *n* | | *n* | |  |  | *n* | | *n* | |  |  |  |
| Participants | 29 | | 25 | |  |  | 18 | | 11 | |  |  | 11 | | 14 | |  |  |  |
|  | *M* | *SD* | *M* | *SD* | *t/U* | *p* | *M* | *SD* | *M* | *SD* | *t/U* | *p* | *M* | *SD* | *M* | *SD* | *t/U* | *p* |  |
| Age in years | 30.21 | 10.76 | 36.36 | 10.02 | 226.5^a^ | .018* | 31.50 | 12.72 | 39.82 | 11.25 | 58.0^a^ | .065 | 28.09 | 6.44 | 33.64 | 8.36 | -1.88 | .074 |  |
| C in ng/ml | 134.03 | 31.27 | 132.14 | 45.79 | .18 | .859 | 121.99 | 28.05 | 121.46 | 41.22 | .04 | .967 | 153.73 | 26.69 | 140.53 | 48.90 | .80 | .430 |  |
| E in pg/ml | 68.23 | 41.97 | 62.89 | 37.31 | 342.0^a^ | .722 | 82.10 | 47.64 | 79.18 | 51.17 | 96.0^a^ | .893 | 45.53 | 12.75 | 50.09 | 12.46 | -.90 | .380 |  |
| T in ng/ml | 1.86 | 1.85 | 2.54 | 2.07 | 295.0^a^ | .242 | 0.46 | 0.16 | 0.42 | 0.21 | .54 | .596 | 4.14 | 0.55 | 4.20 | 1.04 | -.17 | .866 |  |
| *Note.*  NAP: Non-autistic participants; AP: Autistic participants; C: Cortisol; E: Estradiol; T: Testosterone.  ^a^ No normal distribution. Significance of parametric and non-parametric methods were not consistent. Results are based on Mann-Whitney-U-test.  * Indicates *p* < .05; ** indicates *p* <.01. | | | | | | | | | | | | | | | | | | | |

**Table S23**

| *Backwards selection process for the regression models of IGT scores* | | | | | | | |
| --- | --- | --- | --- | --- | --- | --- | --- |
| Group | Model | | Sum of Squares | df | Mean Square | F | Sig. |
| NAP | 1 | Regression | 4912.106 | 5 | 982.421 | 2.173 | .094^b^ |
|  |  | Residual | 9946.608 | 22 | 452.119 |  |  |
|  |  | Total | 14858.714 | 27 |  |  |  |
|  | 2 | Regression | 4864.931 | 4 | 1216.233 | 2.799 | .050^c^ |
|  |  | Residual | 9993.783 | 23 | 434.512 |  |  |
|  |  | Total | 14858.714 | 27 |  |  |  |
|  | 3 | Regression | 4579.031 | 3 | 1526.344 | 3.564 | .029^d^ |
|  |  | Residual | 10279.684 | 24 | 428.320 |  |  |
|  |  | Total | 14858.714 | 27 |  |  |  |
|  | 4 | Regression | 3481.375 | 2 | 1740.687 | 3.825 | .036^e^ |
|  |  | Residual | 11377.340 | 25 | 455.094 |  |  |
|  |  | Total | 14858.714 | 27 |  |  |  |
| AP | 1 | Regression | 6442.047 | 5 | 1288.409 | 1.072 | .407^f^ |
|  |  | Residual | 22832.193 | 19 | 1201.694 |  |  |
|  |  | Total | 29274.240 | 24 |  |  |  |
|  | 2 | Regression | 6204.101 | 4 | 1551.025 | 1.345 | .288^g^ |
|  |  | Residual | 23070.139 | 20 | 1153.507 |  |  |
|  |  | Total | 29274.240 | 24 |  |  |  |
|  | 3 | Regression | 5452.687 | 3 | 1817.562 | 1.602 | .219^h^ |
|  |  | Residual | 23821.553 | 21 | 1134.360 |  |  |
|  |  | Total | 29274.240 | 24 |  |  |  |
|  | 4 | Regression | 5160.960 | 2 | 2580.480 | 2.354 | .118^i^ |
|  |  | Residual | 24113.280 | 22 | 1096.058 |  |  |
|  |  | Total | 29274.240 | 24 |  |  |  |
|  | 5 | Regression | 2559.868 | 1 | 2559.868 | 2.204 | .151^j^ |
|  |  | Residual | 26714.372 | 23 | 1161.494 |  |  |
|  |  | Total | 29274.240 | 24 |  |  |  |
|  | 6 | Regression | .000 | 0 | .000 | . | .^k^ |
|  |  | Residual | 29274.240 | 24 | 1219.760 |  |  |
|  |  | Total | 29274.240 | 24 |  |  |  |
| Note. IGT: Iowa Gambling Task; NAP: Non-autistic participants; AP: Autistic participants.  a. Dependent Variable: IGT net score | | | | | | | |
| b. Predictors: (Constant), transformed estradiol, age, transformed testosterone, cortisol, sex | | | | | | | |
| c. Predictors: (Constant), transformed estradiol, transformed testosterone, cortisol, sex | | | | | | | |
| d. Predictors: (Constant), transformed estradiol, transformed testosterone, sex | | | | | | | |
| e. Predictors: (Constant), transformed estradiol, sex | | | | | | | |
| f. Predictors: (Constant), transformed estradiol, age, cortisol, sex, transformed testosterone | | | | | | | |
| g. Predictors: (Constant), transformed estradiol, age, sex, transformed testosterone | | | | | | | |
| h. Predictors: (Constant), transformed estradiol, age, transformed testosterone | | | | | | | |
| i. Predictors: (Constant), age, transformed testosterone | | | | | | | |
| j. Predictors: (Constant), age | | | | | | | |
| k. Predictor: (constant) | | | | | | | |

| **Table S24**  *Coefficients of backwards selection process for regression models of IGT scores* | | | | | | | |
| --- | --- | --- | --- | --- | --- | --- | --- |
| Group | Model | | Unstandardized Coefficients | | Standardized Coefficients | t | Sig. |
|  |  |  | B | Std. Error | Beta |  |  |
| NAP | 1 | (Constant) | -47.060 | 33.118 |  | -1.421 | .169 |
|  |  | Sex | 47.005 | 17.504 | .997 | 2.685 | .014 |
|  |  | Age | -.144 | .447 | -.060 | -.323 | .750 |
|  |  | Cortisol | .115 | .176 | .151 | .651 | .522 |
|  |  | Transformed testosterone | 11.750 | 10.589 | .411 | 1.110 | .279 |
|  |  | Transformed estradiol | -11.321 | 5.081 | -.458 | -2.228 | .036 |
|  | 2 | (Constant) | -54.013 | 24.673 |  | -2.189 | .039 |
|  |  | Sex | 47.425 | 17.113 | 1.005 | 2.771 | .011 |
|  |  | Cortisol | .133 | .164 | .175 | .811 | .426 |
|  |  | Transformed testosterone | 11.808 | 10.379 | .413 | 1.138 | .267 |
|  |  | Transformed estradiol | -11.417 | 4.973 | -.461 | -2.296 | .031 |
|  | 3 | (Constant) | -35.972 | 10.605 |  | -3.392 | .002 |
|  |  | Sex | 47.856 | 16.982 | 1.015 | 2.818 | .010 |
|  |  | Transformed testosterone | 15.146 | 9.461 | .530 | 1.601 | .122 |
|  |  | Transformed estradiol | -11.192 | 4.930 | -.452 | -2.270 | .032 |
|  | 4 | (Constant) | -23.040 | 7.082 |  | -3.253 | .003 |
|  |  | Sex | 24.775 | 9.249 | .525 | 2.679 | .013 |
|  |  | Transformed estradiol | -8.848 | 4.852 | -.358 | -1.824 | .080 |
| AP | 1 | (Constant) | 82.461 | 41.274 |  | 1.998 | .060 |
|  |  | Sex | -25.363 | 28.895 | -.368 | -.878 | .391 |
|  |  | Age | -1.646 | .835 | -.472 | -1.972 | .063 |
|  |  | Cortisol | -.074 | .166 | -.097 | -.445 | .661 |
|  |  | Transformed testosterone | -20.678 | 13.485 | -.650 | -1.533 | .142 |
|  |  | Transformed estradiol | 9.346 | 9.959 | .244 | .938 | .360 |
|  | 2 | (Constant) | 73.781 | 35.637 |  | 2.070 | .052 |
|  |  | Sex | -22.102 | 27.385 | -.321 | -.807 | .429 |
|  |  | Age | -1.721 | .801 | -.494 | -2.147 | .044 |
|  |  | Transformed testosterone | -20.107 | 13.152 | -.632 | -1.529 | .142 |
|  |  | Transformed estradiol | 8.489 | 9.573 | .222 | .887 | .386 |
|  | 3 | (Constant) | 58.319 | 29.800 |  | 1.957 | .064 |
|  |  | Age | -1.615 | .784 | -.463 | -2.060 | .052 |
|  |  | Transformed testosterone | -11.258 | 7.203 | -.354 | -1.563 | .133 |
|  |  | Transformed estradiol | 3.850 | 7.592 | .101 | .507 | .617 |
|  | 4 | (Constant) | 57.238 | 29.217 |  | 1.959 | .063 |
|  |  | Age | -1.604 | .770 | -.460 | -2.082 | .049 |
|  |  | Transformed testosterone | -10.834 | 7.033 | -.340 | -1.540 | .138 |
|  | 5 | (Constant) | 34.995 | 26.147 |  | 1.338 | .194 |
|  |  | Age | -1.031 | .694 | -.296 | -1.485 | .151 |
|  | 6 | (Constant) | -2.480 | 6.985 |  | -.355 | .726 |
| Note. IGT: Iowa Gambling Task; NAP: Non-autistic participants; AP: Autistic participants.  a. Dependent Variable: IGT net score | | | | | | | |

| **Table S25**  *Regression models for IGT scores with hierarchical entry based on backwards selection outcome* | | | | | | | |
| --- | --- | --- | --- | --- | --- | --- | --- |
| Group | Model | | Sum of Squares | df | Mean Square | F | Sig. |
| NAP | 1 | Regression | 2240.975 | 2 | 1120.487 | 2.220 | .130^b^ |
|  |  | Residual | 12617.740 | 25 | 504.710 |  |  |
|  |  | Total | 14858.714 | 27 |  |  |  |
|  | 2 | Regression | 3765.201 | 3 | 1255.067 | 2.715 | .067^c^ |
|  |  | Residual | 11093.514 | 24 | 462.230 |  |  |
|  |  | Total | 14858.714 | 27 |  |  |  |
| AP | 1 | Regression | 3504.978 | 2 | 1752.489 | 1.496 | .246^b^ |
|  |  | Residual | 25769.262 | 22 | 1171.330 |  |  |
|  |  | Total | 29274.240 | 24 |  |  |  |
|  | 2 | Regression | 3508.076 | 3 | 1169.359 | .953 | .433^c^ |
|  |  | Residual | 25766.164 | 21 | 1226.960 |  |  |
|  |  | Total | 29274.240 | 24 |  |  |  |
| Note. IGT: Iowa Gambling Task; NAP: Non-autistic participants; AP: Autistic participants.  a. Dependent Variable: IGT net score | | | | | | | |
| b. Predictors: (Constant), age, sex | | | | | | | |
| c. Predictors: (Constant), age, sex, transformed estradiol | | | | | | | |

| **Table S26**  *Coefficients of IGT regression models* | | | | | | | | |
| --- | --- | --- | --- | --- | --- | --- | --- | --- |
| Group | Model | | B | Bootstrap^a^ | | | | |
|  |  |  |  | Bias | Std. Error | Sig. (2-tailed) | BCa 95% Confidence Interval | |
|  |  |  |  |  |  |  | Lower | Upper |
| NAP | 1 | (Constant) | -8.499 | -.827 | 11.737 | .467 | -30.290 | 11.484 |
|  |  | Age | -.325 | .024 | .393 | .411 | -1.057 | .544 |
|  |  | Sex | 17.825 | .376 | 8.924 | .058 | -1.635 | 38.217 |
|  | 2 | (Constant) | -13.743 | -3.682 | 16.052 | .304 | -49.606 | 3.272 |
|  |  | Age | -.332 | .114 | .511 | .466 | -1.137 | 1.435 |
|  |  | Sex | 25.475 | .872 | 10.890 | .039 | 1.833 | 52.740 |
|  |  | Transformed estradiol | -8.880 | -.775 | 5.464 | .095 | -20.053 | -1.135 |
| AP | 1 | (Constant) | 36.742 | -1.891 | 27.649 | .207 | -15.574 | 82.745 |
|  |  | Age | -1.236 | .051 | .704 | .101 | -2.767 | .606 |
|  |  | Sex | 13.038 | -.004 | 14.244 | .373 | -14.961 | 42.362 |
|  | 2 | (Constant) | 36.733 | -2.286 | 29.382 | .222 | -21.458 | 87.433 |
|  |  | Age | -1.232 | .061 | .747 | .120 | -2.842 | .734 |
|  |  | Sex | 12.799 | -.491 | 16.006 | .413 | -15.837 | 43.137 |
|  |  | Transformed estradiol | .414 | .087 | 8.213 | .950 | -18.184 | 17.583 |
| Note. IGT: Iowa Gambling Task; NAP: Non-autistic participants; AP: Autistic participants.  a. Unless otherwise noted, bootstrap results are based on 1,000 bootstrap samples | | | | | | | | |

**Transformation formulas**

**Transformed CRT score**

**Root transformation:**

**√x ref * -1**

**xref =(x∗−1) + a;**

**with x = CGT-Score and**

**a = Maximum of CGT-Score + 1**

**Source: https://novustat.com/statistik-blog/spss-test-auf-normalverteilung-negativ.html**
